# Case report: Pathology, antimicrobial resistance, and molecular characterization of bovine abortion cases caused by *Nocardia farcinica* in Korean native cattle

**DOI:** 10.3389/fvets.2024.1407634

**Published:** 2024-08-21

**Authors:** Eun-Mi Kim, Chi Sun Yun, You-Chan Bae, Hyunkyoung Lee, Bo-Youn Moon, Kichan Lee, Hye-Young Jeoung, Bok-Kyung Ku, Jongho Kim

**Affiliations:** Animal Disease Diagnostic Division, Animal and Plant Quarantine Agency, Gimcheon, Republic of Korea

**Keywords:** abortion, antimicrobial resistance, bovine, multilocus sequence analysis, *Nocardia farcinica*

## Abstract

**Introduction:**

*Nocardia farcinica* is an opportunistic bacterium that causes bovine mastitis and pulmonary, cutaneous, and central nervous system infections in humans. Bovine abortion caused by *N. farcinica* has been sporadically reported. The purpose of this study was to analyze the pathological findings of bovine abortions caused by *N. farcinica* in the Republic of Korea and determine the antimicrobial resistance and genotypical characteristics of *N. farcinica* isolates.

**Case presentation:**

Three cases of bovine abortions were submitted to the Animal and Plant Quarantine Agency for differential diagnosis. Grossly, one fetus showed severe lung consolidation following palpation of the entire lobes. Histologically, necrotizing granulomatous interstitial pneumonia was observed in all fetuses; a fetus with a gross lesion demonstrated necrotizing lymphadenitis in the mesenteric lymph nodes and necrotizing dermatitis in the ear. *N. farcinica* isolates were isolated from the abomasal contents and lungs of all fetuses. Finally, two cases were diagnosed as abortions due to *N. farcinica*, and one was diagnosed as an *N. farcinica* abortion coinfected with bovine viral diarrhea virus. According to the multilocus sequence analysis, all isolates were identified as *N. farcinica* and were determined to be genetically related to isolates from humans. Two *N. farcinica* isolates were resistant to trimethoprim-sulfamethoxazole, which is recommended as the first treatment for human nocardial infections.

**Conclusion:**

This is the first pathological report of bovine abortion caused by *N. farcinica* in the Republic of Korea. Further studies are needed to phenotypically and genotypically characterize *N. farcinica* isolates with various sources and continuously monitor antimicrobial resistance patterns.

## 1 Introduction

*Nocardia farcinica* is a ubiquitous saprophyte in the environment and an opportunistic pathogen of humans and animals. Pulmonary, cutaneous, and central nervous system infections caused by *N. farcinica* have been reported in humans (1–3), and *N. farcinica* was a common pathogen in human nocardiosis in Europe and Iran (4, 5) between 2009 and 2015. However, bovine mastitis has been reported as the most common clinical manifestation of nocardiosis, and several sporadic cases of *N. farcinica*-induced bovine abortions have been reported (6–9). Although cases of nocardiosis caused by *N. farcinica* in humans have been documented in the Republic of Korea (KOR), little information is available regarding animal infections caused by *N. farcinica* (10–15).

The most significant treatment aspect for nocardiosis is the selection of appropriate antimicrobials (11). According to previous studies, *N. farcinica* is resistant to cefepime, ceftriaxone, clarithromycin, and tobramycin; intermediate to doxycycline, imipenem, and minocycline; and susceptible to amikacin, amoxicillin-clavulanic acid, ciprofloxacin, linezolid, moxifloxacin, and trimethoprim-sulfamethoxazole. Among these antimicrobials, amikacin, linezolid, and trimethoprim-sulfamethoxazole are appropriate treatments for *Nocardia* spp. infection (16). In particular, trimethoprim-sulfamethoxazole is the preferred initial treatment for patients infected with *N. farcinica* (10, 11).

Although 16S rRNA gene (16S) sequencing of *Nocardia* spp. was considered the gold standard for bacterial identification, the method fails to discriminate among species (17). Multilocus sequence analysis (MLSA) of sequences concatenated with 5–7 housekeeping genes—such as 16S, *gyrB*, *secA1*, *hsp65*, *recA*, *trpB*, *rpoA*, and *rpoB*—is highly discriminative (18, 19), and MLSA helps identify new species, as well as *Nocardia* spp. (18).

Several studies of bovine mastitis caused by *N. farcinica* have reported antimicrobial resistance and/or genetic characterization (20, 21). This study aimed to analyze pathological lesions of bovine nocardial abortion cases, antimicrobial resistance patterns of *N. farcinica* isolates, and genetic characteristics between *N. farcinica* isolates from bovine abortions and humans.

## 2 Case presentation

In January 2022, three bovine aborted fetuses (Bov-fet-1 to Bov-fet-3) from 6- and 7-month pregnant cows (Korean native cattle, *Bos taurus coreanae*) were submitted to the Animal and Plant Quarantine Agency (APQA) from different cities (Jangsu-gun, Gyeongju-si, and Imsil-gun) for differential diagnosis by veterinarians and animal owners, with their respective consent. Therefore, the requirement for ethical approval from the Institutional Animal Care and Use Committee of APQA was waived. Except for abortions, the dams did not show any clinical signs. The gestational age was estimated based on the crown-rump length (22). Full necropsies, including macroscopic examination, were performed in all fetuses. Samples of the brain (cerebrum, cerebellum, and brainstem), tongue, lungs, heart, liver, spleen, kidneys, skeletal muscle, small and large intestines, endocrine organs, and mesenteric lymph nodes were collected for histologic examination and identification of infectious agents. The samples were fixed in 10% neutral buffered formalin and routinely processed with hematoxylin and eosin staining.

Samples of the lungs and abomasal contents were aseptically inoculated on 5% sheep blood (Asan Pharm. Co., Ltd., Seoul, Republic of Korea) and MacConkey (Becton, Dickinson and Company, Franklin Lakes, New Jersey, United States) agars and incubated aerobically at 37°C up to 7 days. The bacterial colonies were transferred to a new 5% sheep blood agar for pure culture. Purely cultured colonies were identified using matrix-assisted laser desorption ionization time-of-flight mass spectrometry (MALDI-TOF MS; bioMérieux, Marcy-l’Étoile, France). All isolates were stored in the BRIX Microvials system (BASIC SCIENCE, Korea) at −80°C until subsequent experiments.

DNA and RNA were separately extracted from the collected samples (brain, tongue, lungs, heart, liver, spleen, kidneys, skeletal muscle, small and large intestines, mesenteric lymph nodes, and endocrine organs) using the Maxwell RSC instrument (Promega, Madison, Wisconsin, United States) with the Maxwell^®^ RSC Blood DNA Kit (Promega) and RSC Viral TNA (Promega), according to the manufacturer’s recommendations.

Polymerase chain reaction (PCR) identifications of major bacterial agents, including *Brucella* spp., *Coxiella burnetii*, *Campylobacter fetus*, *Listeria monocytogenes*, *Leptospira* spp., *Yersinia pseudotuberculosis*, and *Chlamydia abortus*, were performed using a Mastercycler ep gradient S (Eppendorf, Hamburg, Germany) with the extracted DNA from the collected samples as previously described (23). PCRs for viral agents—such as bovine viral diarrhea virus (BVDV), bovine herpesvirus type 1, and five arboviruses, including Akabane virus, Ainovirus, Chuzan virus, bovine ephemeral fever virus, and Ibaraki virus—were performed using the LiliF BVDV real-time RT-PCR kit (iNtRON Biotechnology, Republic of Korea), the LiliF IBR PCR kit (iNtRON Biotechnology), the VDx Bovine Akabane/Aino MP RT-PCR kit (Median Diagnostics, Republic of Korea), and VDx Bovine Chuzan/BEF/Ibaraki MP RT-PCR kit (Median Diagnostics) according to the manufacturer’s instructions. Protozoal (*Neospora caninum*) agents were determined using PCR according to a previous study (24).

Genomic DNA was extracted using the QIAamp DNA mini kit (Qiagen, Hilden, Germany) according to the manufacturer’s instructions. The amplification of 16S, *gyrB*, *secA1*, *hsp65*, and *rpoB* was performed according to previously described methods (Supplementary Table S1). All PCR products were directly sequenced using the Macrogen sequencing service (Macrogen, Republic of Korea). The obtained sequences were deposited in GenBank under the accession numbers ON239083–ON239085 for 16S and ON244769–ON244771 for *rpoB*.

The sequences from the three cases were aligned using the BioEdit version 7.2 program,^1^ and their genetic homology with global *N. farcinica* references in the National Center for Biotechnology Information data was compared. The sequences of 16S, *gyrB*, *secA1*, and *hsp65*—selected according to a previous study—were concatenated in the order *gyrB*-16S-*secA1*-*hsp65* (19). To illustrate the phylogenetic relationship among the *Nocardia* species, each *gyrB*, 16S, *secA1*, and *hsp6* sequence of 35 *Nocardia* species and 16 *N. farcinica* strains was downloaded from the GenBank DataBase and concatenated in the same order as mentioned above (18). The phylogenetic analysis was performed using the MEGA version XI (Pennsylvania State University, State College, Pennsylvania, United States) software based on the concatenated sequences (1,340 bp). Phylogenetic trees based on the two genes (16S and *rpoB*) and MLSA were constructed using the Jukes–Cantor model of the neighbor-joining method with 1,000 bootstrap replicates (10).

Antimicrobial susceptibility tests of *N. farcinica* isolates were performed using a 96-well Sensititre RAPMYCOI plate (Thermo Fisher Scientific, Waltham, Massachusetts, United States) according to the manufacturer’s instructions. *Escherichia coli* ATCC 25922 and *Enterococcus faecalis* ATCC 29212 were used as quality controls (5). Each of the values was interpreted as susceptible (S), intermediate (I), or resistant (R) according to a previous study from the Clinical and Laboratory Standards Institute guideline (25).

## 3 Diagnostic assessment

**Figure 1:**
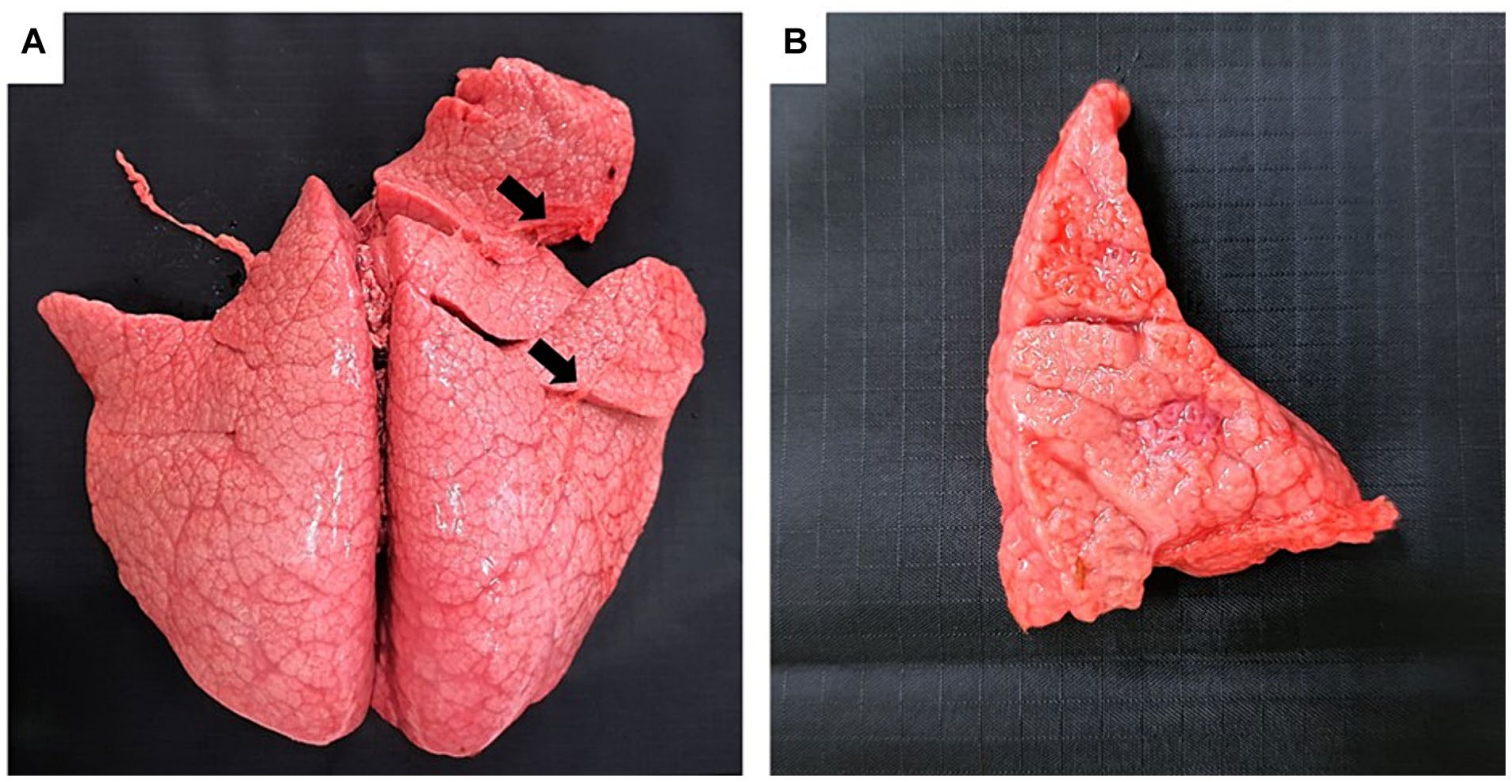
Gross findings of *Nocardia farcinica* abortion in a bovine fetus. **(A)** Bov-fet-1. Lungs. Severely consolidated lungs following palpation of the entire lobes (black arrows). **(B)** Bov-fet-1. Lungs. Cut section of severely consolidated lungs.

**Figure 2:**
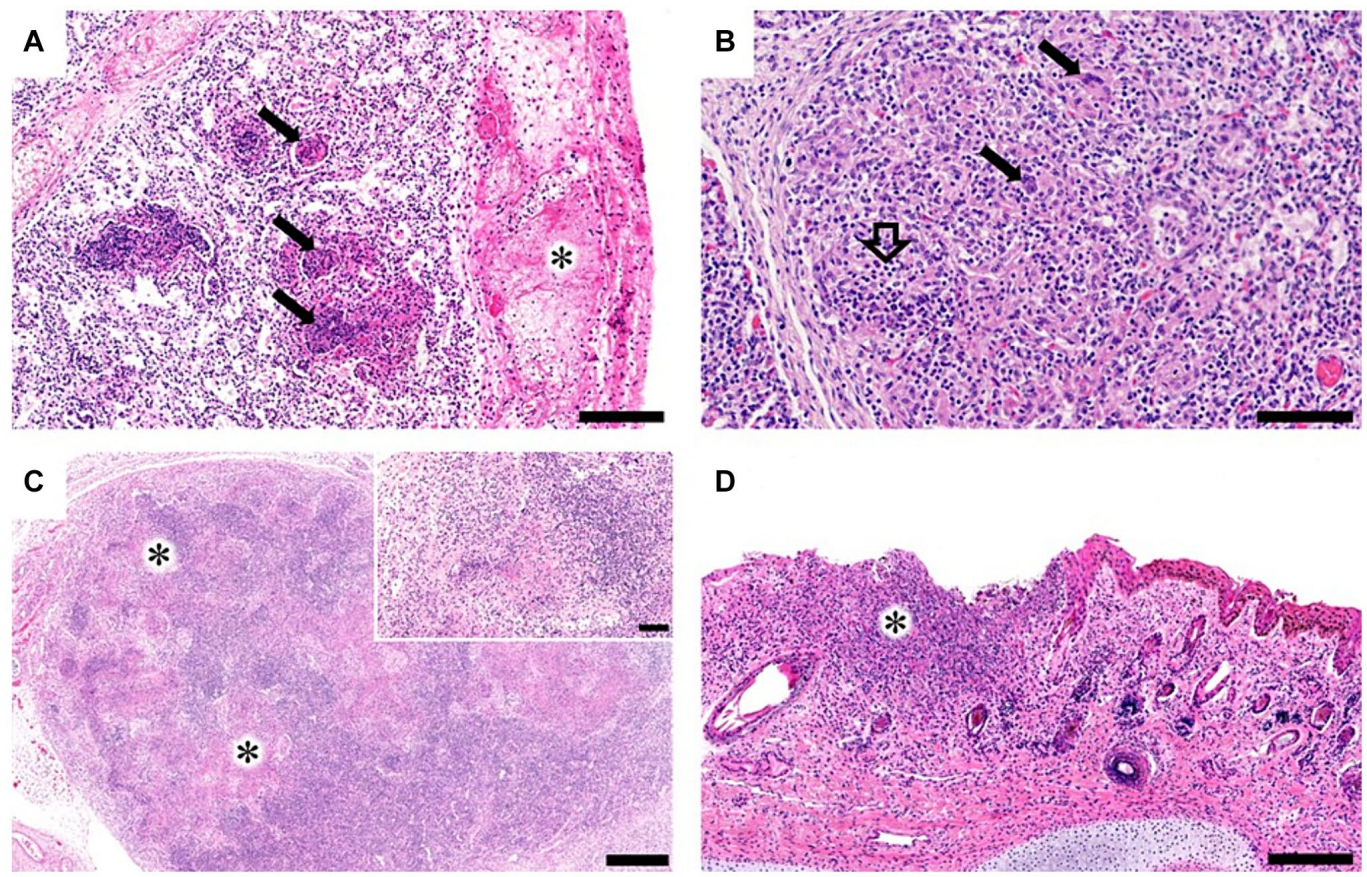
Histopathological findings of bovine aborted fetuses caused by *Nocardia farcinica*. **(A)** Bov-fet-1. Lungs. Multifocal necrosis with inflammatory cells, primarily including macrophages and neutrophils within the alveolar spaces (black arrows) and fibrinous pleuritis (asterisk). H&E stain; scale bar, 200 μm. **(B)** Bov-fet-2. Lungs. Infiltration of macrophages and neutrophils (black hollow arrow) in the alveolar lumen with multinucleated giant cells (black arrows). H&E stain; scale bar, 100 μm. **(C)** Bov-fet-1. Mesenteric lymph nodes. Multifocal necrosis (asterisks, inset). H&E stain; scale bar, 500 μm; inset scale bar, 100 μm. **(D)** Bov-fet-1. Ears. Severe necrosis and infiltration of inflammatory cells, including macrophages, within the dermis (asterisk). H&E stain; scale bar, 200 μm.

Grossly, only Bov-fet-1 had severe lung consolidation following palpation of the entire lobes (Figures 1A,B). Histologically, necrotizing granulomatous interstitial pneumonia with multinucleated giant cells was observed in all fetuses (Figure 2A,B). Bov-fet-1 revealed fibrinous pleuritis, necrotizing lymphadenitis, and dermatitis (Figures 2A,C,D). There were no histologic lesions in Bov-fet-2 and Bov-fet-3, except for those in the lungs.

Except for the case of Bov-fet-2 detected with BVDV, other samples were negative for bacterial, viral, and protozoal agents related to bovine abortion using PCR. *Nocardia*-suspected colonies were isolated from the lungs and abomasal contents of all fetuses. The isolates were identified to be *N. farcinica* using MALDI-TOF MS.

Our isolates were genetically identical for *gyrB*, 16S, *secA1*, *hsp65*, and *rpoB*. Phylogenetic trees (Supplementary Figures S1A,B) based on the partial 16S and *rpoB* genes were constructed for comparison between our isolates and other strains.

In the phylogenetic tree of the partial 16S gene, sequences were compared with eight *Nocardia* spp. in humans using the 16S sequences (Supplementary Figure S1A). These sequences were separated from other *Nocardia* spp. Our isolates were closely associated with strains from brain abscess samples of humans in the KOR and Iran (Supplementary Figure S1A). Compared with our sequences, all *N. farcinica* isolates were identified to have 99.5–100% homology (Supplementary Figure S1A). A phylogenetic tree constructed with the partial *rpoB* gene was compared with eight *Nocardia* spp. in humans (Supplementary Figure S1B). In the *N. farcinica* strains from humans in China, PUNC023 isolated from sputum and PUNC014 isolated from the lungs revealed 98 and 99% identity, respectively, compared with our isolates (Supplementary Figure S1B).

**Figure 3:**
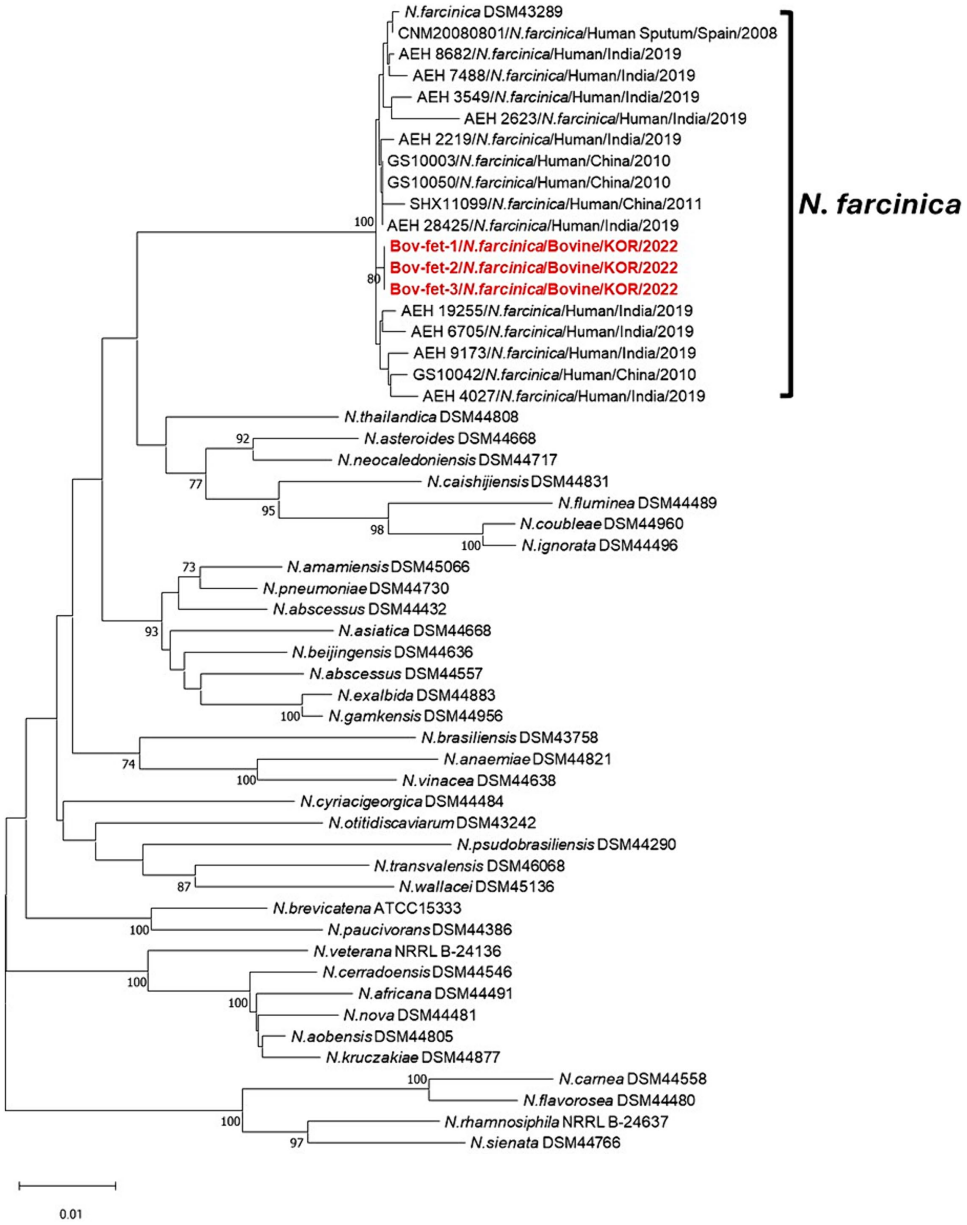
Phylogenetic relationships using concatenated sequence *gyrB*-16S-*secA1*-*hsp65* compared with 35 other *Nocardia* spp. strains and 16 *N. farcinica* isolates, including those from bovine abortion. Phylogenetic analysis was performed using the neighbor-joining method with 1,000 bootstrap replicates. Bootstrap values ≥70% are shown at the nodes. The isolates in this study are represented in red and bold. The criteria for genetic distance are shown at the bottom of the figure.

The concatenated sequences (*gyrB*-16S-*secA1*-*hsp65*) for MLSA were assessed to investigate phylogenetic relationships of *N. farcinica* isolates in the present study (Figure 3). Compared with each sequence of the *N. farcinica* reference strain DSM 43289, 16S and *hsp65* of *N. farcinica* isolates in this study showed 100% sequence identity, and over 99% correspondence was found in *gyrB* and *secA1* (data not shown). The neighbor-joining tree showed that all three samples were clustered to *N. farcinica* isolates, with *N. farcinica* strains from humans in Spain, India, and China. Furthermore, all *N. farcinica* isolates in this study showed close genetic associations with *N. farcinica* strains from humans.

The results of antimicrobial resistance of *N. farcinica* isolates are presented in Table 1. All *N. farcinica* isolates from the three bovine cases showed similar antimicrobial susceptibility patterns. Among the 15 antimicrobials, all isolates were susceptible to amikacin and linezolid, with intermediate resistance to moxifloxacin, minocycline, and amoxicillin-clavulanic acid. All isolates were resistant to the remaining nine antimicrobials, including cefepime, ceftriaxone, cefoxitin, ciprofloxacin, clarithromycin, doxycycline, imipenem, tigecycline, and tobramycin. Additionally, two isolates (Bov-fet-1 and Bov-fet-3) showed antimicrobial resistance to trimethoprim-sulfamethoxazole.

**Table 1:** MIC ranges of *Nocardia farcinica* isolates of the three bovine aborted fetuses.

Antimicrobials	Isolated *N. farcinica* (MIC μg/mL)			MIC (μg/mL) for category (26)			Susceptibility
	Bov-fet-1	Bov-fet-2	Bov-fet-3	Susceptible	Intermediate	Resistant	
Amikacin	≤1	≤1	≤1	≤8	—	≥16	S
Amoxicillin-clavulanic acid	16	16	16	≤4	8	≥16	R
Cefepime	>32	>32	>32	≤8	16	≥32	R
Ceftriaxone	>64	>64	>64	≤8	16–32	≥64	R
Cefoxitin	>128	>128	>128	—	—	—	NT^*^
Ciprofloxacin	4	>4	>4	≤1	2	≥4	R
Clarithromycin	>16	>16	>16	≤2	4	≥8	R
Doxycycline	8	16	16	≤1	2–4	≥8	R
Imipenem	>64	>64	>64	≤4	8	≥16	R
Linezolid	4	4	8	≤8	—	—	S
Minocycline	8	8	8	≤1	2–4	≥8	R
Moxifloxacin	2	2	2	≤1	2	≥4	I
Tigecycline	>4	>4	>4	—	—	—	NT
Tobramycin	>16	>16	>16	≤4	8	≥16	R
Trimethoprim-sulfamethoxazole	152	38	76	≤38	—	≥76	R^**^

^*^NT, not determined. ^**^Except for Bov-fet-2. MIC, minimal inhibitory concentration.

## 4 Discussion

According to the pathological and laboratory examination results of the three cases, Bov-fet-1 and Bov-fet-3 were diagnosed with abortion caused by *N. farcinica*; Bov-fet-2 was identified to be coinfected with *N. farcinica* and BVDV. Additional abortion or stillbirth cases did not occur among non-infected dams housed in the same farms. To our knowledge, this is the first pathological report of bovine abortion caused by *N. farcinica* in the KOR. There have been a few reports of bovine abortion cases caused by *N. farcinica* (26, 27) and *N. asteroides* (28, 29). Similar to previous studies in Japan and the USA, granulomatous pneumonia was found in all fetuses (26, 27). Although organs (lungs, ears, and mesenteric lymph nodes) containing histologic lesions in the present study were different from those reported in previous studies in Japan (lungs, liver, spleen, kidneys, lymph nodes, thyroid, adrenal, and tongue) and the USA (lungs, placenta, and kidneys), similar necrotizing granulomatous lesions were identified in the current study (27). According to a previous study, the causes of abortion were suspected to be contamination of the reproductive tract during obstetric manipulation, resulting in placentitis and fetal systemic infection via the amniotic fluid or fetal circulation (27).

According to the molecular analysis using 16S, *rpoB*, and MLSA, the *N. farcinica* isolates in this study were genetically identical to and clustered with *N. farcinica* strains. Although genetic information of *N. farcinica* isolates from animals and humans in the KOR is lacking, we identified a genetic relatedness between *N. farcinica* isolates in humans and bovine. Therefore, the possibility of zoonotic potential between bovine and human *N. farcinica* isolates should be noted considering these genetic associations.

On comparing the antimicrobial susceptibility results of this study with those of previous studies, the results of susceptible antimicrobials, such as amikacin and linezolid, in this study were consistent with the results of *N. farcinica* isolates from previous studies (16, 30). However, two *N. farcinica* isolates in this study showed antimicrobial resistance to trimethoprim-sulfamethoxazole; these were previously reported to be susceptible (16). According to previous studies, the rates of resistance of human strains from Taiwan, Europe, and Iran to trimethoprim-sulfamethoxazole are low (2–8%) (4, 5). To our knowledge, few studies have reported *N. farcinica* isolates resistant to trimethoprim-sulfamethoxazole from animal hosts (20, 21).

Although human-to-human transmission has not been documented, animal-to-human transmission of nocardiosis has been reported in *N. brasiliensis* infections from cats (31). In the KOR, nocardiosis caused by *N. farcinica* was reported in various diseases in humans (10–15). Most cases occurred in people with weakened immune systems, including people who are older, those who are immunocompromised, and those with colon cancer, diabetes mellitus, or kidney transplantation (10, 13–15). There were some limitations in understanding the pathogenesis of *N. farcinica* abortion owing to the unsubmitted placentas and intrauterine swabs to prove the contamination of the reproductive tract. However, through the *N. farcinica* isolation from abomasal contents and the necrotic lesions of ear skin, further studies considering amniotic fluid contamination are required to understand the pathogenesis of the bovine fetal infections caused by *N. farcinica*.

In conclusion, considering the close genetic relatedness of *N. farcinica* isolates between humans and bovines, animal-to-human transmission should not be neglected, and continuous antimicrobial surveillance and analysis using *N. farcinica* isolates, particularly those resistant to trimethoprim-sulfamethoxazole, will be required to improve public health. Further studies are needed to analyze the genetic characteristics of various *N. farcinica* isolates using more powerful tools for genetic analysis such as whole-genome sequencing (32) and to investigate antimicrobial resistance gene profiles using *N. farcinica* isolates from animals and humans.

## Data availability statement

The datasets presented in this study can be found in online repositories. The names of the repository/repositories and accession number(s) can be found in the article/Supplementary material.

## Ethics statement

The animal studies were approved by Animal and Plant Quarantine Agency. The studies were conducted in accordance with the local legislation and institutional requirements. Written informed consent was obtained from the owners for the participation of their animals in this study. Written informed consent was obtained from the participant/patient(s) for the publication of this case report.

## Author contributions

E-MK: Conceptualization, Investigation, Methodology, Writing – original draft, Writing – review & editing. CY: Conceptualization, Data curation, Formal analysis, Investigation, Software, Writing – original draft, Writing – review & editing. Y-CB: Supervision, Writing – review & editing. HL: Supervision, Writing – review & editing. B-YM: Supervision, Writing – review & editing. KL: Supervision, Writing – review & editing. H-YJ: Supervision, Writing – review & editing. B-KK: Funding acquisition, Supervision, Writing – review & editing. JK: Conceptualization, Investigation, Methodology, Validation, Writing – review & editing.

## Footnotes

^1^ http://www.bioedit.software.informer.com
