# Supplementary material for: Case report: Pathology, antimicrobial resistance, and molecular characterization of bovine abortion cases caused by Nocardia farcinica in Korean native cattle
Source: Front Vet Sci. 2024 Aug 21;11:1407634. doi: 10.3389/fvets.2024.1407634 (PMC11373135; doi:10.3389/fvets.2024.1407634)
Supplement: Supplementary file 1 [file Table_1.docx]

Supplementary Material

**Supplementary Table 1. Clinical presentations of three bovine abortion cases caused by *Nocardia farcinica* in the KOR**

| Case | City | CR length (pregnancy period) | Other identified pathogen | Isolated organs |
| --- | --- | --- | --- | --- |
| Bov-fet-1 | Jangsu-gun | 60 cm (7 months) | No | Lung and abomasal contents |
| Bov-fet-2 | Gyeongju-si | 62 cm (7 months) | BVDV^*^ | Lung and abomasal contents |
| Bov-fet-3 | Imsil-gun | 41 cm (6 months) | No | Lung and abomasal contents |

^*^ BVDV, Bovine viral diarrhea virus.

**Supplementary Table 2.** PCR primers of species-specific DNA fragments for detection, 16s rRNA and rpoB for phylogenetic trees, and 5 housekeeping genes for MLSA

| Primer name | Sequence (5´– 3´) | Target gene | Amplification size (bp) | Reference |
| --- | --- | --- | --- | --- |
| Nf1 | CCG CAG ACC ACG CAA C | Species-specific PCR | 409 | 33 |
| Nf2 | ACG AGG TGA CGG CTG C |  |  |  |
| 16S-F3 | CAG GCC TAA CAC ATG CAA GT | 16S | 1248 | 10 |
| 16S-R3 | GGG CGG WGT GTA CAA GGC |  |  |  |
| MF | CGA CCA CTT CGG CAA CCG | *rpoB* | 296 |  |
| MR | TCG ATC GGG CAC ATC CGG |  |  |  |
| E8F | AGA GTT TGA TCC TGG CTC AG | 16S | 462 | 18 |
| 534r | ATT ACC GCG GCT GCT GG |  |  |  |
| Noc-gyrB-F | CTT CGC CAA CAC CAT CAA CAC | *gyrB* | 482 |  |
| Noc-gyrB-R | TGA TGA TCG ACT GGA CCT CG |  |  |  |
| Noc-gyrB-F3 | CGA GGA GGG CTT CCG CGC GG |  |  |  |
| Noc-gyrB-R3 | ATC GAC TGG ACC TCG TTG TTC |  |  |  |
| secA1-F47 | GCG ACG CCG AGT GGA TGG | *secA1* | 445 |  |
| secA1-ConR2 | TTG GCC TTG ATG GCG TTG TTC |  |  |  |
| secA1-F47 | GCG ACG CCG AGT GGA TGG |  |  |  |
| secA1-ConR | GCG GAC GAT GTA GTC CTT GTC |  |  |  |
| Noc-hsp65-F | ACC AAC GAT GGT GTG TCC AT | *hsp65* | 401 |  |
| Noc-hsp65-R | CTT GTC GAA CCG CAT ACC CT |  |  |  |
| Noc-hsp65-F2 | GTT GTC CTG GAG AAG AAG TGG |  |  |  |
| Noc-hsp65-R | CTT GTC GAA CCG CAT ACC CT |  |  |  |
| Noc-rpoB-F2 | CCG CTA CAA GAT CAA CAA GAA GC | *rpoB* | 400 |  |
| Noc-rpoB-R4 | CGS TCS TCG AGG AAG TTG CCG TC |  |  |  |
| Noc-rpoB-F2 | CCG CTA CAA GAT CAA CAA GAA GC |  |  |  |
| Noc-rpoB-R2 | GGC GAC GTA CTC CAT CTC CTC |  |  |  |
| Noc-rpoB-F4 | CCC GCG AGG ACA TCG TCG |  |  |  |
| Noc-rpoB-R4 | CGS TCS TCG AGG AAG TTG CCG TC |  |  |  |
| Noc-rpoB-F5 | CGA GTA CCT GGT GCG YCT GC |  |  |  |
| Noc-rpoB-R5 | TCG ACC GGC GAG TTG GCC TG |  |  |  |
